# Supplementary material for: Physical limits of sea-level rise adaptation in global river deltas
Source: Nat Commun. 2026 Feb 14;17:2760. doi: 10.1038/s41467-026-69517-7 (PMC13018211; doi:10.1038/s41467-026-69517-7)
Supplement: Supplementary file 1 — Supplementary Information [file 41467_2026_69517_MOESM1_ESM.pdf]

Supplementary Information

Physical limits of sea-level rise adaptation in global river deltas

Kiara G Lasch[1*] (k.g.lasch@uu.nl) https://orcid.org/0000-0001-7933-0384
Jaap H Nienhuis[1] (j.h.nienhuis@uu.nl) https://orcid.org/0000-0002-4296-7450
Gundula Winter[2] (gundula.winter@deltares.nl) https://orcid.org/0000-0002-7455-1687
Marjolijn Haasnoot[1,2] (m.haasnoot@uu.nl) https://orcid.org/0000-0002-9062-4698

[1] Faculty of Geosciences, Department of Physical Geography, Utrecht University, P.O. Box 80115, 3508 TC Utrecht, the Netherlands
[2] Deltares, P.O. Box 177, 2600 MH Delft, the Netherlands
* Corresponding author

Contents

Supplementary Text 1. Adaptation strategies (including examples) 2
Supplementary Text 2. Delta polygon extent 4
Supplementary Text 3. Equations and data sources of physical indicators 4
Supplementary Text 4. Support for thresholds selected 10
Supplementary Text 5. Flood risks for global deltas and differences between climate scenarios 11
Supplementary Text 6. Model output comparison 13
Sensitivity analyses of model performance, parameter uncertainty, and set thresholds 13
Literature assessment and model output comparison for 10 deltas 15
References 17

Supplementary Text 1. Adaptation strategies (including examples)

We assess the physical feasibility of adaptation options for the following five adaptation strategies: advance, protect-closed, protect-open, accommodate, and retreat (Supplementary Table 1).

Supplementary Table 1: Description and examples of five adaptation strategies assessed in this study.

Adaptation strategy	Examples
Advance: involves the extension of the coastline seaward to build flood defences and is typically used to create new land for nature and recreation, or urban and industrial developments. In this study, we also include a coastal lagoon between the mainland and the new coastline. A coastal levee is built at the seaward side of the new coastline. Pumps are further installed to pump excess rainwater and river flows across the new coastline.	• The Flevoland polder and the Afsluitdijk pumping station in the Rhine Meuse delta, the Netherlands[1,2]<br>• Advance strategy along the coast of the Netherlands[3,4]<br>• The Great Garuda project for Jakarta city, Indonesia[5]<br>• Reclaimed land used to free up space on the mainland, Singapore[6]<br>• Polder was created using dike rings instead of sand to build new elevated land to lower sand requirements, Singapore[6]<br>• Development of new coastal estates in Eko Atlantic City, Nigeria[7]<br>• Terrebonne basin barrier island in Louisiana[8]
Protect-closed: aims to keep flood waters away by constructing engineered structures, such as levees, along the coastline, which protects the inland areas from the sea. In addition, pumps are installed at the river mouths to pump water from the low-lying areas to the sea.	• Pumps along the IJmuiden mouth in the Rhine Meuse delta, the Netherlands[3]<br>• A series of dikes, floodwalls and pumping stations along stretches of the coast in Louisiana[9]
Protect-open: maintains an open connection with the sea while still protecting the inland areas from sea-level rise (SLR). This is achieved by extending sea level influences upstream by building levees along the coast and rivers. Moreover, storm surge barriers are built at the river mouths along the coast. These barriers remain open for most of the time, but close during storm surge events to mitigate the effects of elevated water levels.	• The Maeslantkering storm surge barrier in South Holland the Rhine Meuse delta, the Netherlands[10]<br>• A series of dikes, barriers and walls along the estuary, as well as the Thames barrier in London, United Kingdom[11,12]<br>• Dikes along the Fraser River, Canada[13]<br>• Floodwalls and the Inner Harbor Navigation canal (IHNC) Lake Borgne Surge Barrier in Louisiana[8]<br>• Dikes along distributaries of the Ganges river in Dhaka, Bangladesh[14]

	• Seawalls, revetments and sand dunes along part of the coast in the Nile Delta, Egypt[15]
Accommodate: adopts a 'living with water' concept. This strategy implies the continued use of at-risk areas, whereby, no attempt is made to prevent flooding. Instead, land use is adjusted to reduce the vulnerability to SLR and associated floods by elevating the urban areas and surrounding land. This approach often aims to mitigate the economic and health costs associated with floods instead of preventing the flood.	• Building raising following SLR in the Mississippi delta, the United States of America[16]<br>• Elevating homes in the Mekong delta, Vietnam [17]<br>• Flood proofing houses and infrastructure in Los Angeles, USA[18]<br>• Tidal river management in parts of the Ganges-Brahmaputra-Meghna delta, Bangladesh[19]<br>• Flood proofing structures in the Riö-Grande delta, United States of America[20]
Retreat: focuses on a planned and permanent relocation of people, assets, and activities to reduce exposure to coastal hazards caused by SLR-induced flooding.	• Climate-driven community retreat on the Isle de Jean Charles, Gulf of Mexico[21]<br>• Voluntary buyouts of flood-prone properties in the Mississippi river valley, United States of America[22]<br>• Permanent retreat from damaged homes and infrastructure in the Greater Toronto Area (GTA), Canada[13]<br>• Forced resettlement programs in the Mekong delta, Vietnam[17]<br>• Household scale resettlement, Vietnam[17]

Supplementary Text 2. Delta polygon extent

The global delta dataset defines deltas as four-point deltaic extents (DN = delta node, RM = river mouth, S1 = shoreline position 1, S2 = shoreline position 2)(Supplementary Fig. 1)[23].

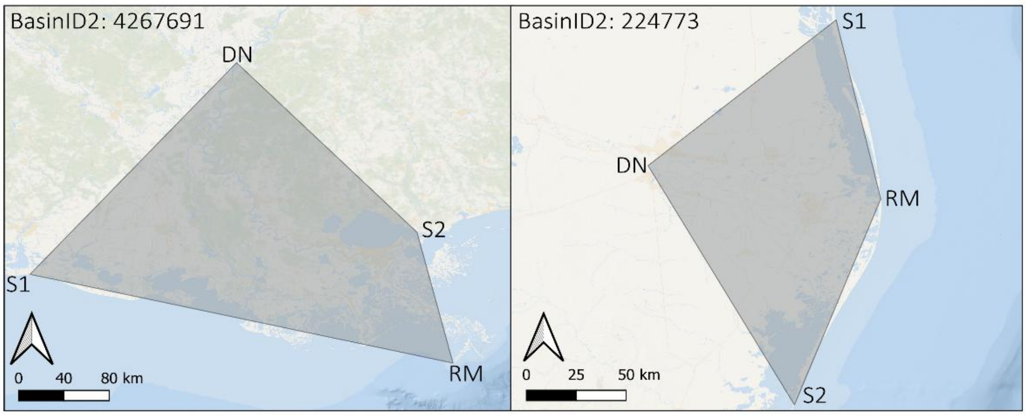

Supplementary Fig. 1: Two examples of deltas defined by the four points of a polygon. DN represents the delta node, S1 and S2 represent the lateral shoreline positions, and RM represents the river mouth[23]. The basemap was created using publicly available ESRI Ocean data (World Ocean Base | ArcGIS Online)[65].

Supplementary Text 3. Equations and data sources of physical indicators

The physical indicators for each adaptation strategy are calculated using the following equations in a simple geometric model:

Advance

The volume of material required (m^3) to extend the coastline seaward of a 2km coastal lagoon, and build a smooth, gentle-sloped (1:6) levee at its seaward edge (Supplementary Fig. 2) is calculated using:

$$V_{offshore} = (d_1 * D_2 * c) + \left(\frac{1}{2} * (d_2 - d_1) * D_2 * c\right)$$

$$h_c = 3 * (H_w + H_{ss})$$

$$b_1 = h_c$$

$$b_2 = b_1 * 6$$

$$V_{coast\ levee} = \left(\frac{1}{2} * (b_1 + b_2) * h_c * c\right) + (RSLR * b_2 * c)$$

$$V_{adv}\,(\mathrm{m^3.s^{-1}}) = V_{offshore} + V_{coast\ levee} \qquad (1)$$

Variable definitions:

- $V_{offshore}$: Total volume of material (m^3) required to aggrade the new coastline offshore
- d_1: offshore depth (m) at 2km from coastline

- d_2: offshore depth (m) at 10km from coastline
- D_2: Distance of 8km offshore
- c: Coastline length (m)
- h_c: Coastal levee height (m)
- H_w: Mean significant wave height (m)[24]
- H_{ss}: Storm surge height (m)[23,25]
- b_1: Short base of the coastal levee (i.e. the top of the levee)
- b_2: Long base of the coastal levee (i.e. the bottom of the levee)
- $V_{coast\ levee}$: Total volume of material (m^3) required to build coastal levees
- RSLR: Relative sea-level rise (m)

Offshore depths (d_1 and d_2) are calculated using the bathymetric slope ($m.m^{-1}$) immediately offshore of the river mouth which is assumed to be linear[24]. The coastline length (c) is calculated as the distance between the coordinates which demarcate the shoreline position S1 and S2 in the delta polygon[23,26] (Supplementary Fig. 1). The significant wave height (H_w) is the average of the largest 1/3 of wave heights using the NOAA WAVEWATCH III 30-year Hindcast Phase 2 between 1979 and 2009[27]. H_{ss} data has a 100-year return-period[23] and is calculated using the median of recorded storm surge values[25]. *RSLR* is the sum of the predicted *SLR* (m) following three climate scenarios, namely SSP1-2.6, SSP2-4.5 and SSP5-8.5[28], and the *VLM* (mm/yr)[29] by 2100. We include subsidence in this equation because omitting it from global SLR risk assessments may underestimate exposure[30].

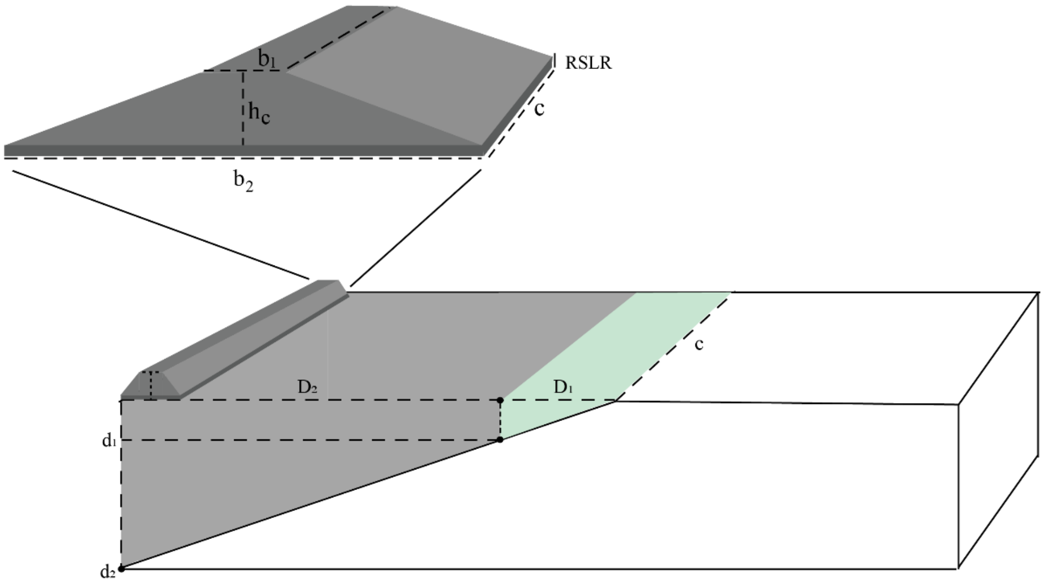

Supplementary Fig. 2: Shape of the volume of material required to extend the coastline seaward based on Eqn. 1. The green area off the coast represents a coastal lagoon.

The total amount of river sediment required (m^3) to advance the coastline seaward beyond the coastal lagoon over 50 years is calculated using:

$$Q_s\,(m^3.s^{-1}) = \frac{Q_s\,(kg.s^{-1})}{\rho_b\,(kg.m^{-3})}$$
$$Sediment\ collected_{adv}\,(m^3) = \int_0^{T_{retention}} (Q_s * R_r)dt \quad (2)$$

Here, Q_s (kg.s^{-1}) is the mean annual river sediment discharge[31,32] which is assumed to remain unchanged until 2100 (see Supplementary Text 6 for sensitivity analysis using projected sediment discharge values). Sediment discharge is converted to m^3.s^{-1} by assuming the bulk density of the sediment (ρ_b) is 1600kg.m^{-3}. We estimate the total volume of sediment collected over a 50-year period ($T_{retention}$) and using different sediment retention rates (R_r, %)(see Methods). Since sediment retention rates vary between 2% and 100% according to existing literature (Supplementary Table 2), we consider three representative retention estimates, namely 20%, 40% and 80% which correspond to low-resource, current known, and innovative thresholds, respectively. If, for example, the volume of river sediment retained at a 40% retention rate exceeds the sediment required to aggrade the coastline seaward, then the measure is considered physically under current known conditions. However, if the volume of river sediment contained at a 20% retention rate is insufficient to meet the sediment demand for the coastline extension, the measure is considered unfeasible under low-resource conditions.

Alternatively, deltas can collect offshore sand as a material source to aggrade a new coastline instead of river sediment. The depth (m) at 10km offshore for sand minding is calculated using the offshore distance (m) and bathymetric slope[24]. Under each climate scenario, we add the SLR value to this depth calculation[28].

The pump capacity (PC)(m^3/s) is calculated using:

$$PC_{mean}(m^3.s^{-1}) = Q_{r,mean}$$
$$PC_{max}(m^3.s^{-1}) = Q_{r,max} \quad (3)$$

Where the pump capacity (PC_{mean} or PC_{max}) is either equal to the mean annual river discharge, ($Q_{r,,mean}$)[31,32], or maximum river discharge ($Q_{r,max}$), respectively, assuming a 100% pump efficiency and that the river discharge will not change by 2100 (see Supplementary Text 6 for sensitivity analysis using projected river discharge values). The maximum river discharge ($Q_{r,max}$) is the 99th percentile of discharges, which is a modelled value from the Water Balance Model (WBM) reanalysis between 1980 and 2012[31].

The required water retention capacity to store excess river discharge during a flood event (maximum river discharge), assuming a 3-day flood event, is calculated using:

$$V_{river,excess} = \int_0^{T_{flood}} (Q_{r,max} - PC_{max})\,dt$$
$$V_{ret,needed} = d(A_{ret,advance} + A_{ret,waterbodies} + A_{ret,wetlands}) \quad (4)$$

Where river discharge during a flood event ($Q_{r,rmax}$) is the 99th percentile of discharges, as above[31], and where T_{flood} is 3 days. We assess the storage capacity of potential retention areas ($V_{ret,needed}$) by summing the area in the advanced delta ($A_{ret,advance}$), calculated as a product of offshore distance (2000m) and coastline length (c)(Supplementary Fig. 2), and permanent water bodies ($A_{ret,waterbodies}$) and wetland areas ($A_{ret,wetlands}$) extracted from the global land use dataset[33]. Each retention area is assumed to have a water storage capacity of d=1m depth.

<u>Protect-closed</u>

The volume of material required (m^3) to build a smooth, gentle-sloped (1:6) coastal levee (Supplementary Fig. 3) is calculated using:

$$h_c = 3 * (H_w + H_{ss})$$
$$b_{1c} = h_c$$
$$b_{2c} = b_{1c} * 6$$
$$V_{coast} = \left(\frac{1}{2} * (b_{1c} + b_{2c}) * h_c * c\right) + (RSLR * b_{2c} * c) \quad (5)$$

Variable definitions:

- h_c: Coastal levee height (m)
- H_w: Mean significant wave height (m)[24]
- H_{ss}: Storm surge height (m)[23,25]
- b_{1c}: Short base of the coastal levee (i.e. the top of the levee)
- b_{2c}: Long base of the coastal levee (i.e. the bottom of the levee)
- V_{coast}: Total volume of material (m^3) required to build coastal levees
- c: Coastline length (m)
- RSLR: Relative sea-level rise (m)

The significant wave height (H_w), storm surge height (H_{ss}) and RSLR are calculated as described above (Eqn. 1).

The mean and maximum pump capacity (PC_{mean} or PC_{max}) is calculated using the same data and equation as discussed above (Eqn. 3).

The required water retention capacity to store excess river discharge during a flood event (maximum river discharge), assuming a 3-day flood event is calculated as described above (Eqn. 4). However, retention areas in a protect-closed strategy only include permanent water bodies ($A_{ret,waterbodies}$) and wetland areas ($A_{ret,wetlands}$) extracted from the global land use dataset[33].

<u>Protect-open</u>

The volume of levee material required (m^3) to build levees (Supplementary Fig. 3) along the coast and both sides of the rivers is calculated using:

$$h_r = 5m = b_{1r}$$
$$b_{2r} = b_{1r} * 6$$

$$V_{river} = 2 * \left(\frac{1}{2} * (b_{1r} + b_{2r}) * h_r * L_r\right) + (RSLR * b_{2r} * L_r)$$
$$h_c = 3 * (H_w + H_{ss})$$
$$b_{1c} = h_c$$
$$b_{2c} = b_{1c} * 6$$
$$V_{coast} = \left(\frac{1}{2} * (b_{1c} + b_{2c}) * h_c * (c - w_r)\right) + (RSLR * b_{2c} * (c - w_r))$$
$$\mathrm{V}_{total} = \mathrm{V}_{coast} + \mathrm{V}_{river} \quad (6)$$

Variable definitions:

- h_r: River levee height (m)
- b_{1r}: Short base of the river levee (i.e. the top of the levee)
- b_{2r}: Long base of the river levee (i.e. the bottom of the levee)
- V_{river}: Total volume of material (m^3) required to build river levees on both sides of the river
- L_r: Total river length (m) [34]
- RSLR: Relative sea-level rise (m)
- h_c: Coastal levee height (m)
- H_w: Mean significant wave height (m)[24]
- H_{ss}: Storm surge height (m)[23,25]
- b_{1c}: Short base of the coastal levee (i.e. the top of the levee)
- b_{2c}: Long base of the coastal levee (i.e. the bottom of the levee)
- V_{coast}: Total volume of material (m^3) required to build coastal levees
- c: Coastline length (m)
- w_r: Width of the river mouth (m)
- V_{total}: Total volume of material (m^3) required to build both coastal and river levees

Here, h_r is the river levee height (m) which we base on existing studies that show that levee heights can vary between 1m and 12m high, and can reach up to 21m[35–37]. We use an average levee height of 5m which excludes the uncommon and extreme cases. We extract the river lengths (L_r) from the Surface Water and Ocean Topography River Database (SWORD) dataset which provides high-resolution river reaches (~10km) and river nodes (200m) at a global scale[34]. Where SWORD is missing a river length (214 cases) within the polygon, we calculate the river length manually. We assume that the river length is equal to the length between the coordinates demarcating the delta node (DN) and the river mouth (RM)(Supplementary Fig. 1). DN, in this case, is the upstream-most bifurcation of the parent channel, and RM is the location of the widest river mouth along the coastline. *RSLR* is the sum of the predicted *SLR* (m) following three climate scenarios, namely SSP1-2.6, SSP2-4.5 and SSP5-8.5[28], and the *VLM* ($mm.yr^{-1}$)[29] by 2100.

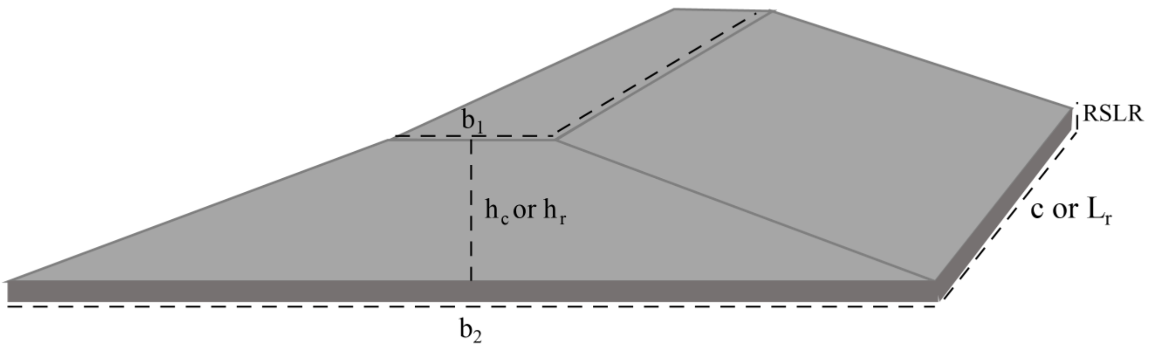

Supplementary Fig. 3: Assumed shape of the levee to calculate the volume of material required for construction, following Eqn. 1, 5 and 6. In the equations, b_{1c} refers to the b_1 of coastal (c) levees, whereas b_{1r} refers to b_1 of river (r) levees. Similarly, h_c refers to the height of the coastal levees, whereas h_r refers to the height of the river levees.

The river width required to build a storm surge barrier (m) is extracted and summed from the SWORD dataset[34]. Where river widths are missing (369 deltas), we calculate these values using a simple river-mouth width (w_m) estimate[38]:

$$w_m = \beta * k * a * L + w_u \quad (7)$$

Here, β = w/d, where *w* the channel width and *d* is the channel depth, *k* is the proportionality coefficient that relates the tidal prism to the cross-sectional area to the river mouth, α is the offshore tidal amplitude (m), L is the estuarine length sale for long-wave propagation in a distributary channel (m) and w_u is the fluvial channel width (m)[38]. The calculated river mouth widths have been compared to observed river mouth widths and show very good agreement, with no systematic bias[38]. However, the calculated river mouth widths tend to be lower than those from SWORD, likely because the river mouth estimate assumes a single channel whereas values from SWORD include multiple river mouths whose combined width can be ~50% greater. However, the river mouth width estimate is used in small deltas, typically with only one distributary mouth.

Accommodate

The 2019 Copernicus global land cover dataset is used to identify land cover within each polygon[33]. This dataset distinguishes 21 land cover types, which we categorize into three main groups, namely nature, cropland, urban (built-up). We isolate urban land use from this dataset, overlay it with ~1km resolution flood maps containing global inundation projections with global mean values that correspond to the climate scenario used[39], and downscale it to 100m resolution. The flood maps in our analysis were created using a static flood modelling approach with extreme sea levels from combined tide and surge levels and accounting for national estimates of flood protection standards. These maps use the Multi-Error-Removed Improved-Terrain (MERIT) digital elevation model. MERIT has previously been found to be consistently higher than the reference, specifically in areas with built-up land cover[40]. This may result in an underestimation of the flooding in urban areas.

The flood depth of each urban land use grid cell is identified and the thresholds are applied, assuming the urban areas can be raised by of 0.5m, 1m or 2m. If the mean flood depth in the urban area, based on the flood maps[39], exceeds 0.5m, then raising by 0.5m is physically unfeasible. Similarly, if the mean flood depth in the urban area exceeds 1m or 2m, then raising by 1m or 2m is physically unfeasible. However, if the flood depth is lower than 1m, then a 1m elevation is considered to be physically feasible.

Retreat

The land availability for a retreat (LA_{ret}) is calculated using (Eqn. 8):

$$LA_{ret} = \frac{A_r\ (m^2)}{A_{uf}\ (m^2)} \quad (8)$$

where A_r represents three areas to retreat to (see methods) and A_{uf} represents the urban flooded area that needs to retreat. LA_{ret} is expressed as a ratio, where values greater than 1 indicate that retreat is physically feasible. Retreat to areas outside the delta is always deemed physically feasible.

Do nothing (no strategy required)

The presence or absence of flood risks in the delta polygon are identified using the flood maps[39]. Where no flood risks were predicted under each climate scenario by 2100, these deltas are assumed to do nothing.

Supplementary Text 4. Support for thresholds selected

The "current known" threshold is determined using existing examples of adaptation measures in literature, and refers to the largest known or most commonly used value of a measure (Supplementary Dataset 1 and Supplementary Table 2). While our indicators' thresholds are based on currently implemented scales of measures, these thresholds may vary based on a delta's capabilities and resources.

Supplementary Table 2: Examples of published natural sediment retention rate estimates in delta plains with references to their literature sources.

Delta, Country	Sediment retention rate (%)	Reference
Amazon, Brazil	41 (over 15 years)	[41–44]
Guadiana, Portugal	2	[45]
Burdekin, Australia	2	[46,47]
Mekong, Vietnam	102	[48–50]
Rhine, the Netherlands	13 to 67	[51,52]
Ob, Russia	43 (over 30 years)	[53]
Yangtze, China	37	[54,55]

The "low-resource" threshold is defined as half the value of the "current known" threshold (see Table 2 in manuscript). We use the relation between income level and adaptation capacity to

support this threshold selection and apply all thresholds globally. We assume that some (lower income) countries would have lower resources than other (higher income) countries, and find that the largest known scales of measures are in higher income countries (on which we base our "current known" thresholds). Specifically, data from Supplementary Dataset 1 shows that home raising and land reclamation efforts in lower-income countries are generally smaller (number of homes and height raised) and less frequent (smaller areas of land over prolonged periods of time) than in higher-income countries by approximately a factor of 2. As such, we believe that the 2-fold difference in thresholds between the low-resource and current-known thresholds are conservative, but validated against existing examples.

The "innovative" threshold is defined as twice the value of the "current known" threshold (see Table 2 in manuscript). This threshold reflects the importance of scaling-up measures for long term sustainability[56]. While there are currently no projections for how each adaptation technology will evolve by 2100, there have been significant increases in technological and infrastructural capabilities. For example, flood management practices have advanced between 2000 and 2017, from costly and basic structural flood control measures that impact biodiversity, to environmentally friendly adaptation strategies that build resilience and enable rapid recovery[57]. For example, in the Netherlands, flood defences have evolved from the Afsluitdijk (1932) and the Delta Works with storm surge barriers like the Eastern Scheldt (1986) and Maeslant Barrier (1997) to recent adaptive and nature-based projects such as the Room for the River and the Sand Motor[58]. Other examples of technological developments over longer timescales include innovation in infrastructural capabilities, which has led to an increase of over 10-fold in building height between 1995 and 2008[59]. Similarly, the volume of material extracted from dredging has increased by nearly 10-fold from 1925 to 2015[60]. This reflects the substantial scale of technological innovations in the last century. However, the scales of other technologies, such as levee heights or storm surge barrier widths, have changed less over the last 100 years. As such, assuming a 2-fold increase in technological capabilities by 2100 is a realistic pace of innovation, albeit conservative for certain measures.

For strategies like accommodate where homes are raised by more than 1m, or retreat where people and assets are relocated outside of the delta, such technologies to implement these measures at "innovative" scales already exist (Supplementary Dataset 1) but have not been implemented delta-wide, which would require innovation. Similarly, while one storm-surge barrier of 9km has already been constructed (Supplementary Dataset 1), constructing multiple barriers of similar scale would also require innovation in terms of resources, space, and planning. Thus, the innovative threshold not only represents possible physical limits of technology (in the case of pump capacity), but also the application of measures at a larger scale (accommodate or protect-open) and the coordination required for their implementation.

Supplementary Text 5. Flood risks for global deltas and differences between climate scenarios

Our data shows that all 769 global deltas will experience sea-level rise following each climate scenario (Mean = 0.48m under SSP1-2.6; Mean = 0.6m under SSP2-4.5; Mean = 0.94m under

SSP5-8.5). Additionally, at least 79% of global deltas will experience flooding under a 100-year return storm surge event. This increases to 82% and 86% under higher climate scenarios (SSP2-4.5 and SSP5-8.5), respectively.

However, when comparing the number of physically feasible adaptation strategies for deltas across climate scenarios, we find that the differences between scenarios are minor (1.62% decrease; Supplementary Fig. 4). Instead, the thresholds applied to the adaptation measures have a greater influence on the number of strategies that are physically feasible (35% increase; Supplementary Fig. 4). For only one delta, namely the Rhine-Meuse delta in the Netherlands, there are no physically feasible low-resource strategies across all three climate scenarios (Supplementary Fig. 4). In this delta, only current known scales of measures or innovative solutions are physically feasible to be adopted delta-wide given the deltas large physical characteristics, large urban area, and large flooded extent.

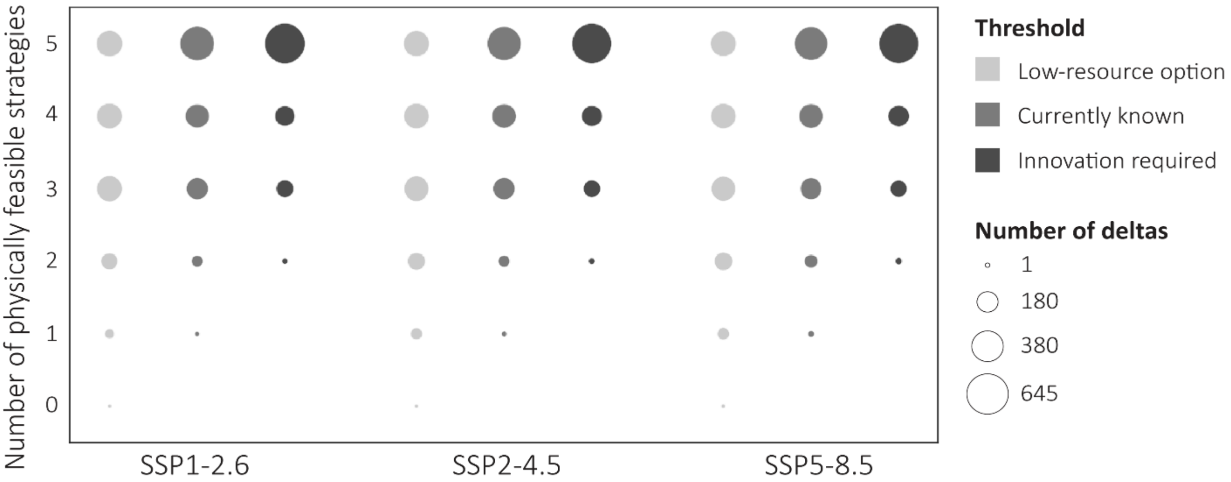

Supplementary Fig. 4: The number of physically feasible adaptation strategies (between 0 and 5) for global deltas following three climate scenarios. The size of the bubbles represent the number of deltas that can choose between each range of adaptation options. The coloured thresholds represent different scales of adaptation measures, namely low-resource measures that are physically feasible under limited resource conditions, current known measures that are the largest known examples of measures or commonly used scales of measures, and innovative measures which are only physically feasible with technological advancements.

In the future, high-end estimates find that sea-levels may rise to between 0.9m (by 2100) and 2.5m (by 2300) following an SSP1-2.6 scenario or between 1.6m (by 2100) and 10.4m (by 2300) following an SSP5-8.5 scenario[61]. Our sensitivity analysis reveals that 2m and 4m of SLR constrain the PSS for many deltas, specifically for the advance, retreat, and accommodate strategies (Supplementary Fig. 5). Higher sea levels means larger offshore depths for dredging and land aggrading (advance), less available non-flooded land (retreat), and greater infrastructure elevation requirements following larger flood depths (accommodate) which constrains the physical feasibility of implementing these strategies.

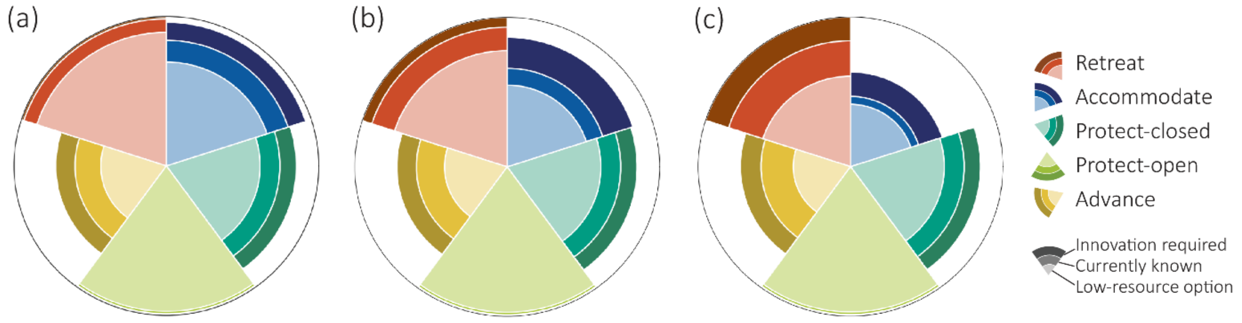

Supplementary Fig. 5: Radar plots comparing (a) the physical solution space (PSS) of global deltas under an SSP2-4.5 scenario and the outcomes of the sensitivity analysis with (b) 2m and (c) 4m of future SLR.

Supplementary Text 6. Model output comparison

Sensitivity analyses of model performance, parameter uncertainty, and set thresholds

To validate the model performance under extreme conditions, we perform a sensitivity test by increasing or decreasing parameters by an order of magnitude well beyond plausible ranges (± 10 or ×/÷ 10)(Supplementary Fig. 6). This confirms the expected expansion of the PSS when the delta's physical characteristics are small, and the contraction of the PSS when the delta's physical characteristics are large. This stress test serves as a boundary check which illustrates model reliability rather than reflecting parameter uncertainty.

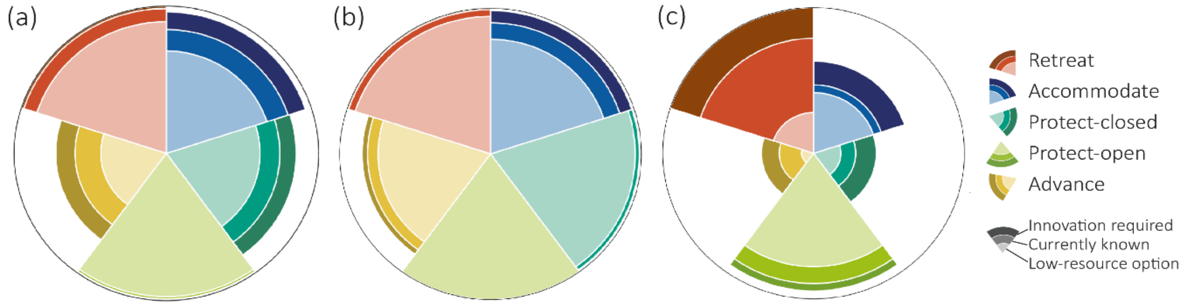

Supplementary Fig. 6: Radar plots comparing (a) the physical solution space (PSS) of global deltas under an SSP2-4.5 scenario, with the outcomes from a stress-test of the model by (b) decreasing or (c) increasing input parameters well beyond plausible ranges to assess how the PSS contracts or expands, respectively.

We also assess parameter uncertainty by performing a sensitivity analysis. Based on projected changes in river discharge, mean flow is expected to vary across river basins, from a 23% decrease to a 65% increase[62]. Globally, mean river discharge is projected to increase by 2%, 6%, 7.5%, and 11% under RCP2.6, 4.5, 6.0, and 8.5 scenarios, respectively, by the end of this century[63]. In contrast, projected sediment discharge for many deltas around the world shows a reduction in sediment flux, with mean declines of approximately 38% by 2100[62], while the mean global sediment flux is projected to increase by 11%, 15%, 14%, and 16.4% across the four emission scenarios[64]. We use these projections to test the sensitivity of our input

parameters on the physical feasibility of strategies. Specifically, we vary river discharge between −23% and +65% and sediment flux between −38% and +16% to consider the full range of variability from both basin and global scale projections. Under increased river discharge projections, protect-closed becomes physically constrained in 66 deltas that could previously adopt it under static conditions. Increased future river discharges raise pump capacity requirements that exceed current capabilities, and expand the required retention areas for excess discharge storage, thereby constraining the PSS of these deltas. Examples include the Incomati (Mozambique), Wami (Tanzania) and Noatak (Alaska) deltas which are in regions (East Africa and the Arctic, respectively) that are projected to increase according to previous studies[62,63]. Under these future conditions, innovation, hybrid, or alternative adaptation strategies may be required to adapt to future flood risks. Under combined increased river and sediment discharge projections, the number of deltas that can adopt advance decreases by 51 deltas compared to no sediment or river discharge change. Despite higher sediment loads to aggrade the coastlines, increased river discharges constrain the pump capacity capabilities and decrease the overall physical feasibility of this strategy. Examples include the Jiulong Jiang (China) and Labuk (Malaysia) deltas, which are both located in tropical (monsoon) regions where river and sediment discharges are projected to increase[62,63].

Under decreased river discharge projections, we find that 40 additional deltas can adopt a protect-closed strategy given lower pump capacity requirements and lower discharges during extreme events which decreases the amount of space required to retain excess river water. For example, in regions where river discharges are projected to decrease, such as Southern Europe and North America[62,63], the PSS for protect-closed is projected to expand, as found in the Ebro (Spain) and the Altamha (USA) deltas, respectively. Under both decreased river and sediment discharge projections, the advance strategy becomes physically feasible for an additional 27 deltas, primarily due to reduced pump capacity requirements. In these deltas, the pump capacity plays a larger role in determining physical feasibility of advance than availability of river sediment to aggrade the coast, given that alternative material sources such as offshore sand remain accessible despite decreased sediment discharges. Examples include the Altamha (USA) and Kikori (Papua New Guinea) deltas, where river and sediment discharges are projected to decrease, consistent with previous studies reporting these trends for North America and Australia, respectively[62,63]. While projected increases or decreases of these parameters impacts several individual deltas which has implications for local scale decision-making, the general adaptation trends remain mostly consistent across the global scale.

We test the sensitivity of assuming 5m high river levees following the protect-open strategy by changing this height and recalculating the material requirements (2m = 8.4km^3; 5m = 14.56km^3; 10m = 33.64km^3). We find that the overall message remains the same whereby the protect-open strategy has higher material requirements than protect-closed strategy, even when considering lower-end levee heights.

Finally, we explore the influence of the chosen innovative threshold on the PSS. We increase the threshold by an order of magnitude, as opposed to a twofold increase, and find that substantial innovation in technological capabilities does not necessarily imply more strategies

are physically feasible. While certain adaptation measures, such as 10m stilts following an accommodate strategy, or 12,000m^3.s^{-1} pumps following a protect-closed strategy, increase the PSS for some deltas, the PSS of other deltas remain unchanged due to fundamental physical characteristics. This highlights that innovation alone does not provide more adaptation opportunities. Given these findings, we maintain the assumption of a twofold increase in innovation capabilities for our analysis since it is more realistic by 2100, and avoids overestimating adaptation opportunities.

Literature assessment and model output comparison for 10 deltas

The model is tested by first applying the equations and thresholds to 10 field deltas, which vary in size, degree of urbanization, and flood extent (Supplementary Fig. 7; Supplementary Table 3; Supplementary Dataset 2).

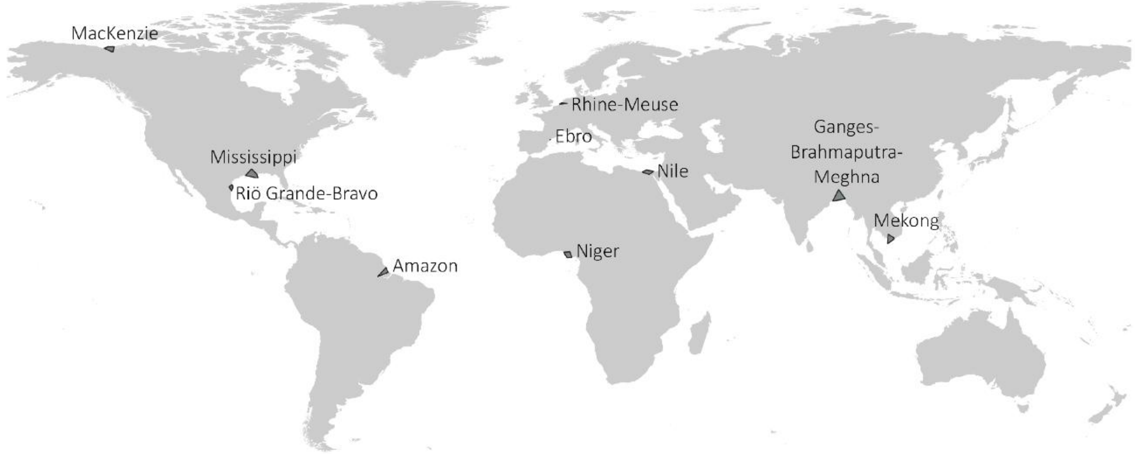

Supplementary Fig. 7: Names and locations of 10 deltas for model testing. Blank basemap was created using publicly available World Countries shapefile data in QGIS (10m Cultural Vectors | Natural Earth Data)[66].

Supplementary Table 3: Delta names and country of 10 deltas for model testing.

Number	Delta name	Country
1	Amazon	South America
2	Ebro	Spain
3	Ganges-Brahmaputra-Meghna	Bangladesh
4	Mekong	Vietnam
5	MacKenzie	Canada
6	Mississippi	United States of America
7	Niger	Nigeria
8	Nile	Egypt
9	Rhine-Meuse	The Netherlands
10	Riö Grande- Bravo	United States of America

We compare our model outputs with literature that focusses on current implemented adaptation measures and potential future strategies in these deltas. The model outcomes are mostly consistent with measures used in practice (Supplementary Dataset 2). For example, in the Ebro delta, future strategies based on literature include protect, advance, or accommodate, which we find to be physically feasible based on our model (Supplementary Dataset 2). Additionally, in the Mississippi delta, future measures to address flood risks include relocations within the delta, land raising or elevations of urban areas, and protective measures, which we also find to be physically feasible strategies in 2100 (Supplementary Dataset 2).

However, there are also measures in the literature that are not consistent in the PSS that we modelled. This may be primarily because we assessed the PSS assuming the strategy will be adopted across the entire delta, however, small scale, localized strategies may also be implemented in these deltas. For example, in the Ganges-Brahmaputra-Meghna delta, the modelled PSS is small, but according to literature, many other measures are already implemented in this delta at a smaller scale (Supplementary Dataset 2). Additionally, in some cases, the hazards that measures are implemented to be effective against or the currently implemented measures based on literature are not measures that we assess within the adaptation strategy, as seen for the Ganges-Brahmaputra-Meghna and the Amazon delta, respectively (Supplementary Dataset 2).

References

1. Rijkswaterstaat. The Afsluitdijk. https://www.rijkswaterstaat.nl/en/projects/iconic-structures/the-afsluitdijk.
2. Hoeksema, R. J. Three stages in the history of land reclamation in the Netherlands. *Irrigation and Drainage* **56**, (2007).
3. van Alphen, J., Haasnoot, M. & Diermanse, F. Uncertain Accelerated Sea-Level Rise, Potential Consequences, and Adaptive Strategies in The Netherlands. *Water (Switzerland)* **14**, (2022).
4. Van Alphen, J., Haasnoot, M., Diermanse, F. & Nillesen, A. L. Beyond the Limits of Present Adaptation Strategies: Exploring Strategies and Measures to Anticipate on Accelerated Sea-Level Rise in the Netherlands. *J Coast Zone Manag* **27**, 7 (2024).
5. Colven, E. Understanding the Allure of Big Infrastructure: Jakarta's Great Garuda Sea Wall Project. *Water Alternatives* **10(2)**, 250–264 (2017).
6. Channel News Asia (CNA). Singapore uses non-traditional method to create new land at Pulau Tekong. https://www.channelnewsasia.com/watch/singapore-uses-non-traditional-method-create-new-land-pulau-tekong-5338486 (2025).
7. Ajibade, I. Can a future city enhance urban resilience and sustainability? A political ecology analysis of Eko Atlantic city, Nigeria. *International Journal of Disaster Risk Reduction* **26**, 85–92 (2017).
8. State of Louisiana. *Louisiana's Comprehensive Master Plan for a Sustainable Coast: Executive Summary 4th Edition*. https://coastal.la.gov/wp-content/uploads/2023/05/230418_CPRA_Executive-Summary_final.pdf (2023).
9. U.S. Army Corps of Engineers. Algiers Canal Risk Reduction Features. https://www.mvn.usace.army.mil/Portals/56/docs/HSDRRS/Hurricane%20%20Storm%20Damage%20Risk%20Reduction%20System%20Fact%20Sheets.pdf (2012).
10. De Bruijn, K. M., Diermanse, F. L. M., Weiler, O. M., De Jong, J. S. & Haasnoot, M. Protecting the Rhine-Meuse delta against sea level rise: What to do with the river's discharge? *J. Flood Risk Manag.* **15**, (2022).
11. Lavery, S. & Donovan, B. Flood risk management in the Thames Estuary looking ahead 100 years. *Philosophical Transactions of the Royal Society A: Mathematical, Physical and Engineering Sciences* **363**, 1455–1474 (2005).
12. Lumbroso, D. & Ramsbottom, D. Flood Risk Management in the United Kingdom: Putting Climate Change Adaptation Into Practice in the Thames Estuary. *Resilience* 79–87 (2018) doi:10.1016/B978-0-12-811891-7.00006-2.
13. Doberstein, B., Fitzgibbons, J. & Mitchell, C. Protect, accommodate, retreat or avoid (PARA): Canadian community options for flood disaster risk reduction and flood resilience. *Natural Hazards* **98**, 31–50 (2019).
14. Rahman, M. A. & Islam, S. Climate Change Adaptation in Urban Areas: A Critical Assessment of the Structural and Non-structural Flood Protection Measures in Dhaka.

in *Confronting Climate Change in Bangladesh* 161–173 (Springer, Cham, 2019). doi:10.1007/978-3-030-05237-9_11.

15. Sharaan, M., Iskander, M. & Udo, K. Coastal adaptation to Sea Level Rise: An overview of Egypt's efforts. *Ocean Coast. Manag.* **218**, (2022).

16. Erdman, J. A., Williams, E. A., James, C. W. & Coakley, G. P. Raising Buildings: The Resilience of Elevated Structures. in *Mississippi Delta Restoration: Pathways to a sustainable future* 143–170 (Springer, Cham, 2018). doi:10.1007/978-3-319-65663-2_10.

17. Garschagen, M. Risky Change? Vietnam's Urban Flood Risk Governance between Climate Dynamics and Transformation. *Pac. Aff.* **88**, 599–621 (2015).

18. Aerts, J. C. J. H. *et al.* Pathways to resilience: adapting to sea level rise in Los Angeles. *Ann. N. Y. Acad. Sci.* **1427**, 1–90 (2018).

19. Islam, M. F., Middelkoop, H., Schot, P. P., Dekker, S. C. & Griffioen, J. Enhancing effectiveness of tidal river management in southwest Bangladesh polders by improving sedimentation and shortening inundation time. *J. Hydrol. (Amst).* **590**, 125228 (2020).

20. U.S. Army Corps of Engineers. Coastal Texas Protection and Ecosystem Restoration Feasibility Study: Final Environmental Impact Statement. https://www.swg.usace.army.mil/Portals/26/Coastal%20Texas%20Protection%20and%20Ecosystem%20Restoration%20Feasibility%20Study_2021FEIS_1.pdf (2021).

21. Simms, J. R. Z., Waller, H. L., Brunet, C. & Jenkins, P. The long goodbye on a disappearing, ancestral island: a just retreat from Isle de Jean Charles. *J. Environ. Stud. Sci.* **11**, 316–328 (2021).

22. Magnan, A. K. *et al.* Status of global coastal adaptation. *Nat. Clim. Chang.* **13**, 1213–1221 (2023).

23. Edmonds, D. A., Caldwell, R. L., Brondizio, E. S. & Siani, S. M. O. Coastal flooding will disproportionately impact people on river deltas. *Nat. Commun.* **11**, (2020).

24. Caldwell, R. L. *et al.* A global delta dataset and the environmental variables that predict delta formation on marine coastlines. *Earth Surface Dynamics* **7**, 773–787 (2019).

25. Muis, S., Verlaan, M., Winsemius, H. C., Aerts, J. C. J. H. & Ward, P. J. A global reanalysis of storm surges and extreme sea levels. *Nature Communications 2016 7:1* **7**, 1–12 (2016).

26. Nienhuis, J. H., Cox, J. R., O'Dell, J., Edmonds, D. A. & Scussolini, P. A global open-source database of flood-protection levees on river deltas (openDELvE). *Natural Hazards and Earth System Sciences* **22**, 4087–4101 (2022).

27. Chawla, A., Spindler, D. M. & Tolman, H. L. Validation of a thirty year wave hindcast using the Climate Forecast System Reanalysis winds. *Ocean Model. (Oxf).* **70**, 189–206 (2013).

28. Fox-Kemper, B. *et al.* Ocean, Cryosphere and Sea Level Change. in *Climate Change 2021: The Physical Science Basis. Contribution of Working Group I to the Sixth Assessment Report of the Intergovernmental Panel on Climate Change* (eds. Masson-Delmotte, V. et al.) 1211–1362 (Cambridge University Press, Cambridge, United Kingdom and New York, NY, USA, 2021). doi:10.1017/9781009157896.011.

29. Oelsmann, J. *et al.* Regional variations in relative sea-level changes influenced by nonlinear vertical land motion. *Nat. Geosci.* **17**, 137–144 (2024).

30. Nicholls, R. J. *et al.* A global analysis of subsidence, relative sea-level change and coastal flood exposure. *Nat. Clim. Chang.* **11**, 338–342 (2021).

31. Cohen, S., Kettner, A. J., Syvitski, J. P. M. & Fekete, B. M. WBMsed, a distributed global-scale riverine sediment flux model: Model description and validation. *Comput. Geosci.* **53**, 80–93 (2013).

32. Nienhuis, J. H. *et al.* Global-scale human impact on delta morphology has led to net land area gain. *Nature* **577**, 514–518 (2020).

33. Buchhorn, M. *et al.* Copernicus Global Land Service: Land Cover 100m: collection 3: epoch 2018: Globe. https://zenodo.org/records/3518038 (2019).

34. Altenau, E. H. *et al.* The Surface Water and Ocean Topography (SWOT) Mission River Database (SWORD): A Global River Network for Satellite Data Products. *Water Resour. Res.* **57**, e2021WR030054 (2021).

35. Lim, M. Seven years after tsunami, Japanese live uneasily with seawalls. https://www.reuters.com/article/us-japan-disaster-seawalls/seven-years-after-tsunami-japanese-live-uneasily-with-seawalls-idUSKCN1GL0DK/ (2018).

36. Teramura, J. & Shimatani, Y. Advantages of the Open Levee (Kasumi-Tei), a Traditional Japanese River Technology on the Matsuura River, from an Ecosystem-Based Disaster Risk Reduction Perspective. *Water 2021, Vol. 13, Page 480* **13**, 480 (2021).

37. Koelewijn, A., Pol, J. & van Schaijk, M. Performance of flood defences in the Netherlands duringthe 2021 summer floods. *Journal of Coastal and Riverine Flood Risk* **2**, (2023).

38. Nienhuis, J. H., Hoitink, A. J. F. T. & Törnqvist, T. E. Future Change to Tide-Influenced Deltas. *Geophys. Res. Lett.* **45**, 3499–3507 (2018).

39. Haasnoot, M. *et al.* Long-term sea-level rise necessitates a commitment to adaptation: A first order assessment. *Clim. Risk Manag.* **34**, 100355 (2021).

40. Pronk, M. *et al.* DeltaDTM: A global coastal digital terrain model. *Scientific Data 2024 11:1* **11**, 1–18 (2024).

41. Dunne, T. , Mertes, L. A. K. , Meade, R. H. , Richey, J. E. , & Forsberg, B. R. Exchanges of sediment between the flood plain and channel of the Amazon River in Brazil. *Bulletin of the Geological Society of America* **110(4)**, 450–467 (1998).

42. Nittrouer, C. A. & DeMaster, D. J. Sedimentary processes on the Amazon continental shelf: past, present and future research. *Cont. Shelf Res.* **6**, 5–30 (1986).

43. Nittrouer, C. A. *et al.* Amazon Sediment Transport and Accumulation along the Continuum of Mixed Fluvial and Marine Processes. *Ann. Rev. Mar. Sci.* **13**, 501–536 (2021).

44. Rine, J. M. & Ginsburg, R. N. Depositional facies of a mud shoreface in Suriname, South America; a mud analogue to sandy, shallow-marine deposits. *Journal of Sedimentary Research* **55**, 633–652 (1985).

45. Morales, J. A. Evolution and facies architecture of the mesotidal Guadiana River delta (S.W. Spain-Portugal). *Mar. Geol.* **138**, 127–148 (1997).

46. Fielding, C. R., Trueman, J. & Alexander, J. Sedimentology of the Modern and Holocene Burdekin River Delta of North Queensland, Australia—Controlled by River Output, not by Waves and Tides. in *River Deltas-Concepts, Models, and Examples* 467–496 (SEPM (Society for Sedimentary Geology), 2005). doi:10.2110/PEC.05.83.0467.

47. Fielding, C. R., Trueman, J. D. & Alexander, J. Holocene Depositional History of the Burdekin River Delta of Northeastern Australia: A Model for a Low-Accommodation, Highstand Delta. *Journal of Sedimentary Research* **76**, 411–428 (2006).

48. Van Nguyen, L., Ta, T. K. O. & Tateishi, M. Late Holocene depositional environments and coastal evolution of the Mekong River Delta, Southern Vietnam. *J. Asian Earth Sci.* **18**, 427–439 (2000).

49. Ta, T. K. O. *et al.* Holocene delta evolution and sediment discharge of the Mekong River, southern Vietnam. *Quat. Sci. Rev.* **21**, 1807–1819 (2002).

50. Xue, Z., Liu, J. P., DeMaster, D., Van Nguyen, L. & Ta, T. K. O. Late Holocene Evolution of the Mekong Subaqueous Delta, Southern Vietnam. *Mar. Geol.* **269**, 46–60 (2010).

51. Middelkoop, H., Erkens, G. & van der Perk, M. The Rhine delta-a record of sediment trapping over time scales from millennia to decades. *J. Soils Sediments* **10**, 1–12 (2010).

52. Erkens, Gilles. Sediment dynamics in the Rhine catchment: Quantification of fluvial response to climate change and human impact. *Utrecht University* 278 (2009).

53. Bobrovitskaya, N. N. & Meade, R. H. Discharges and yields of suspended sediment in the Ob' and Yenisey Rivers of Siberia. in *IAHS Publications-Series of Proceedings and Reports-Intern Assoc Hydrological Sciences* vol. 236 115–124 (1996).

54. Hori, K., Saito, Y., Zhao, Q. & Wang, P. Architecture and evolution of the tide-dominated Changjiang (Yangtze) River delta, China. *Sediment. Geol.* **146**, 249–264 (2002).

55. Liu, J. P. *et al.* Flux and fate of Yangtze River sediment delivered to the East China Sea. *Geomorphology* **85**, 208–224 (2007).

56. Guerriero, R. & Penning-Rowsell, E. C. Innovation in flood risk management: An 'Avenues of Innovation' analysis. *J. Flood Risk Manag.* **14**, e12677 (2021).

57. Wang, L. *et al.* A review of the flood management: from flood control to flood resilience. *Heliyon* **8**, (2022).

58. Rijkswaterstaat. The sand motor. www.dezandmotor.nl (2015).

59. Kayvani, K. The evolution of tall buildings: past and present trendsends. https://www.aurecongroup.com/insights/tall-buildings-past-and-present-trends.

60. Cooper, A. H., Brown, T. J., Price, S. J., Ford, J. R. & Waters, C. N. Humans are the most significant global geomorphological driving force of the 21st century. *Anthropocene Review* **5**, 222–229 (2018).

61. van de Wal, R. S. W. *et al.* A High-End Estimate of Sea Level Rise for Practitioners. *Earths Future* **10**, e2022EF002751 (2022).

62. Van Vliet, M. T. H. *et al.* Global river discharge and water temperature under climate change. *Global Environmental Change* **23**, 450–464 (2013).

63. Moragoda, N. & Cohen, S. Climate-induced trends in global riverine water discharge and suspended sediment dynamics in the 21st century. *Glob. Planet. Change* **191**, 103199 (2020).

64. Dunn, F. E. *et al.* Projections of declining fluvial sediment delivery to major deltas worldwide in response to climate change and anthropogenic stress. *Environmental Research Letters* **14**, 084034 (2019).

65. ArcGIS REST Services Directory. Ocean. World Ocean Base. https://services.arcgisonline.com/arcgis/rest/services/Ocean/World_Ocean_Base/MapServer.

66. Natural Earth Data. 1:10m Cultural Vectors . https://www.naturalearthdata.com/downloads/10m-cultural-vectors/.
