# Supplementary material for: Physical limits of sea-level rise adaptation in global river deltas
Source: Nat Commun. 2026 Feb 14;17:2760. doi: 10.1038/s41467-026-69517-7 (PMC13018211; doi:10.1038/s41467-026-69517-7)
Supplement: Supplementary file 2 — Description of Additional Supplementary Files [file 41467_2026_69517_MOESM2_ESM.pdf]

Description of Additional Supplementary Files

Supplementary Dataset 1: Database of existing examples of measures within adaptation strategies and references to literature. These measures represent the physical indicators in our assessment and the recorded magnitudes are used to create the respective thresholds.

Supplementary Dataset 2: Comparison between the calculated physical solution space in this assessment, and the existing implemented or future strategies in 10 deltas based on existing literature.
